# Combined Effect of Inflammation and Hyperglycemia on Mild Cognitive Impairment and Associated Dietary Patterns in an Older Taiwanese Population

**DOI:** 10.3389/fnut.2022.791929

**Published:** 2022-02-18

**Authors:** Yen-Chun Fan, Chia-Chi Chou, Bagas Suryo Bintoro, Wen-Harn Pan, Chyi-Huey Bai

**Affiliations:** ^1^School of Public Health, College of Public Health, Taipei Medical University, Taipei, Taiwan; ^2^Institute of Epidemiology and Preventive Medicine, National Taiwan University, Taipei, Taiwan; ^3^School of Medicine, Chang Gung University, Taoyuan, Taiwan; ^4^Department of Internal Medicine, Chang Gung Memorial Hospital, Keelung, Taiwan; ^5^International Master/Ph.D. Program in Medicine, College of Medicine, Taipei Medical University, Taipei, Taiwan; ^6^Department of Health Behavior, Environment, and Social Medicine, Faculty of Medicine, Public Health and Nursing, Universitas Gadjah Mada, Yogyakarta, Indonesia; ^7^Center of Health Behavior and Promotion, Faculty of Medicine, Public Health and Nursing, Universitas Gadjah Mada, Yogyakarta, Indonesia; ^8^Institute of Biomedical Sciences, Academia Sinica, Taipei, Taiwan; ^9^Department of Public Health, College of Medicine, Taipei Medical University, Taipei, Taiwan; ^10^Nutrition Research Center, Taipei Medical University Hospital, Taipei, Taiwan

**Keywords:** joint effect, CRP, HbA1c, cognitive function, dietary pattern

## Abstract

**Background:**

Previous studies have demonstrated that C-reactive protein (CRP) and glycated hemoglobin (HbA1c) levels are independently associated with neurodegenerative diseases, which can be improved by altering dietary patterns. This study investigates the combined effect of CRP and HbA1c, as well as the influence of dietary patterns, on the risk of dementia.

**Methods:**

A cross-sectional study was conducted with 536 participants aged ≥65 years who were recruited from the Nutrition and Health Survey in Taiwan between 2014 and 2016. The high levels of inflammation and glycation were defined as a CRP level of >0.21 mg/dl and a HbA1c level of ≥6.50%, respectively. Mild cognitive impairment (MCI) was evaluated using the Mini-Mental State Examination (MMSE) score. The dietary patterns associated with CRP and HbA1c levels were assessed using the reduced rank regression (RRR). Multivariate logistic regression analysis of both complete and imputed datasets was performed.

**Results:**

Participants with high levels of both CRP and HbA1c were associated with the highest odds ratio (*OR*) of MCI (adjusted *OR* [a*OR*] = 3.52; 95% *CI* = 3.48, 3.56; *p* < 0.001), followed by a high level of only HbA1c (a*OR* = 1.73; *p* < 0.001) and a high level of CRP (a*OR* = 1.49; *p* < 0.001). Using the reduced rank regression, an inverse relationship between higher consumption nuts and seeds and lower levels of CRP and HbA1c was found (both factors loading < −0.2). Concerning the combined effect of tertiles among the factor 1 and factor 2 analyzed by dietary patterns, group 1 with both T3 (high tertiles) was associated with the greatest *OR* of MCI (a*OR* = 4.38; 95% *CI* = 4.34, 4.42; *p* < 0.001) using multiple imputation.

**Conclusions:**

The combined effect of high levels of inflammation and hyperglycemia was associated with an increased likelihood of MCI. Moreover, dietary patterns positively related to inflammation and hyperglycemia were associated with MCI, while eating nuts and seeds promoted better cognition.

## Introduction

The incidence and prevalence of dementia and Alzheimer's disease (AD) have increased in recent years (1, 2), leading to critical socioeconomic burdens (3, 4) that will inevitably worsen in the future as the population ages (5, 6). Therefore, it is crucial to understand the potential factors affected by cognitive impairment.

Among cardiovascular risk factors, the main factor leading to the development of dementia is diabetes mellitus (DM) due to the pathophysiology of insulin resistance (IR) (7, 8). Glycated hemoglobin (HbA1c) might be treated as a surrogate marker or diagnostic criterion for the glycemic status that is related to IR in DM (9, 10), which still does not completely explain its presentation in DM (11, 12). In addition, uncontrolled inflammation may result in the progression of neurodegenerative diseases (NGDs) through damage to brain neurons; however, a well-controlled inflammatory status could slow the process (13). A strong positive correlation between high-sensitivity C-reactive protein (hs-CRP) levels and HbA1c levels was noted in patients with type 2 DM (14).

Exploring the enhanced effects of both inflammation and glycation on the development of mild cognitive impairment (MCI) might provide clinically relevant insights. Previous studies have shown that the combined effects of HbA1c and hs-CRP are associated with advanced subclinical carotid atherosclerosis progression (15), cardiovascular risks (16), and coronary artery diseases (17). However, no studies have examined the association between the combined effect of glycated status and inflammation on the risk of cognitive dysfunction. Furthermore, glycemic control due to dietary improvements was demonstrated to be beneficial for patients with type 2 DM (18). However, inconsistent relationships between inflammatory biomarkers and dietary patterns have been reported (19). Therefore, it would be helpful to identify particular dietary patterns that decrease the adverse impacts of inflammation and hyperglycemia.

Using a nationwide population-based survey, this study investigates the combined effect of glycemic metabolic disorders and inflammation, expressed as high levels of CRP and HbA1c, on MCI. To determine the dietary patterns related to inflammation and hyperglycemia, we attempted to explore dietary patterns related to higher CRP and HbA1c levels and to express the effects of dietary patterns and particular food groups on MCI.

## Methods

### Study Design and Subjects

A cross-sectional study was performed using the Nutrition and Health Survey in Taiwan (NAHSIT) between 2014 and 2016. A nationwide population-based survey is conducted on a regular yearly basis to explore the relationships between dietary nutritional patterns and MCI in non-institutionalized Taiwanese people. Demographic data regarding the sociodemographic characteristics, lifestyle factors, self-reported medical history, and dietary intake were obtained through face-to-face interviews conducted by well-trained personnel. Subsequently, the physical examination information included anthropometric data, and biochemical parameters were collected at a temporary health examination station. Detailed information regarding the characteristics of the participants and the research design of recruitment for the NAHSIT have been previously described (20, 21).

**Figure 1:**
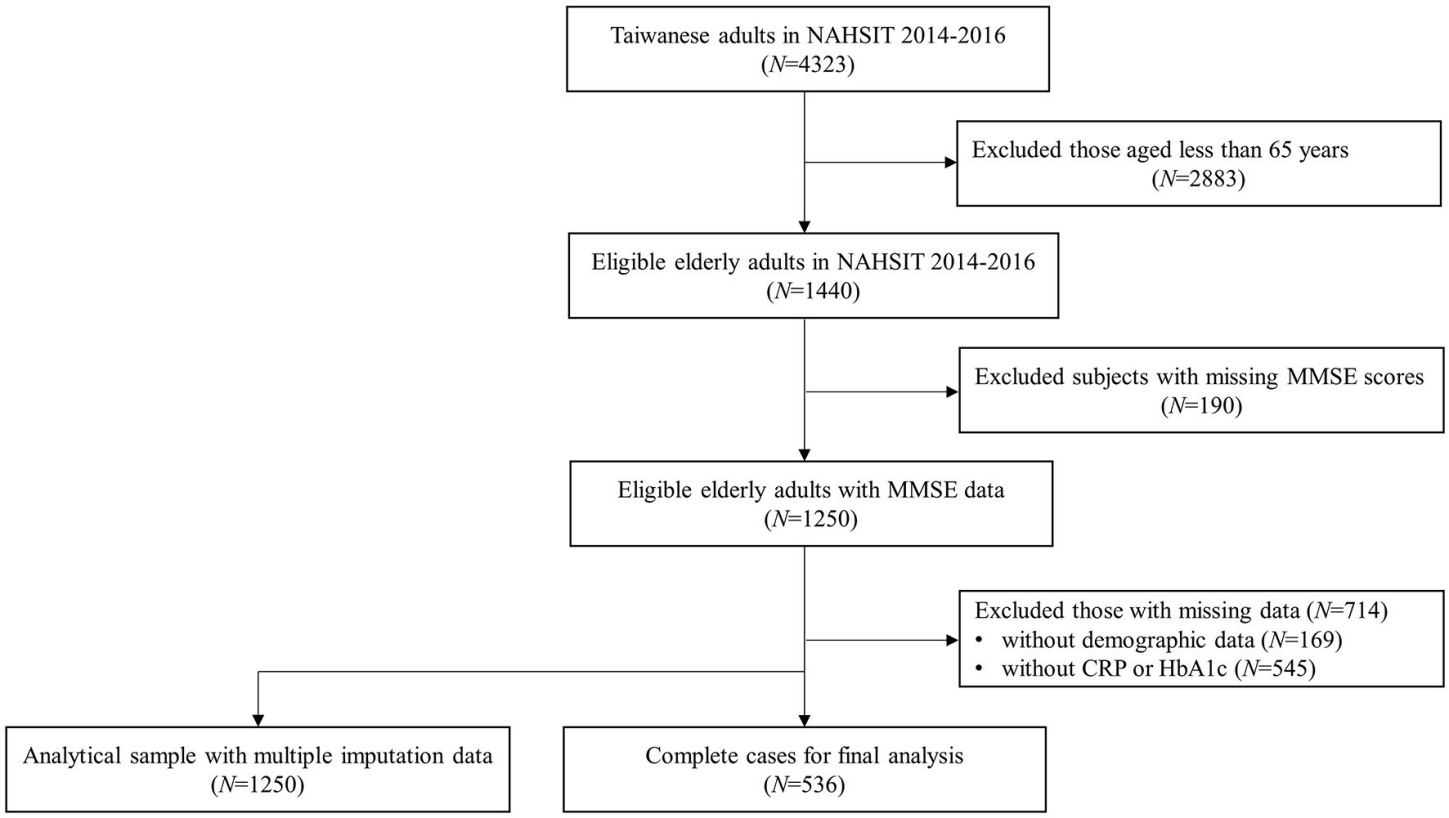
The flowchart of participants' selection.

A total of 4,323 participants were recruited from NAHSIT between 2014 and 2016. Among them, 1,440 eligible older adults aged ≥65 years were included in the study. After excluding those without the Mini-Mental State Examination (MMSE) scores, the remaining 1,250 eligible older adults with MMSE data were obtained. Those with missing demographic data (*N* = 169) and biochemical indicators of CRP and HbA1c (*N* = 545) were also excluded. Finally, a total of 536 older adults aged 65 years and older were included in the final analysis. Additionally, those with missing data were then imputed to construct an analytical sample of 1,250 older participants. A flowchart of the participants' selection is presented in Figure 1. The Joint Institutional Review Board of Taipei Medical University reviewed and approved the study.

### Definition of High Levels of CRP and HbA1c

In NAHSIT 2014–2016, the biomarker of inflammation was only available for CRP but not for high-sensitivity C-reactive protein (hs-CRP). The biomarker of HbA1c was tested by high-performance liquid chromatography (HPLC); additionally, the particle-enhanced immunoturbidimetric test was used to detect the level of CRP. During the period between 2014 and 2016, 8-h fasting blood samples were obtained from the participants to measure the CRP and HbA1c concentrations.

High levels of inflammation and glycation were defined as a CRP level of >0.21 mg/dl (22) and a HbA1c level of ≥6.50% (23), respectively, in the final sample and in the analytical sample with multiple imputation. Therefore, the study sample was divided into three groups based on the status of CRP and HbA1c: participants with high levels of both inflammation and glycation (IG, *N* = 43), participants with high levels of either inflammation or glycation (EIG, *N* = 179), and participants with high levels of neither inflammation nor glycation (NN, *N* = 314). In addition, those with a high level of either inflammation or glycation were subsequently categorized into two groups: participants with high levels of inflammation but not glycation (IN, *N* = 86) and participants without high levels of inflammation but with glycation (NG, *N* = 93).

### Dietary Assessment

The NAHSIT 2014–2016 contained a 79-item food-frequency questionnaire (FFQ) to investigate the dietary information. Detailed information has been previously published (24). This dietary food questionnaire was divided into 23 food groups, such as vegetables, fresh fruits, whole grains, rice, noodle and their products, breakfast cereals, roots and tubers, nuts and seeds, dairies, flavored milk, soybean products, eggs, fish, shellfish, seafood products, poultry, red meat, processed meat products, organ meats, fried foods, snacks, coffee, tea, and sweetened beverages. The reliability of a similar simplified FFQ has been validated in the Elderly Nutrient and Health Survey in Taiwan (25). Moreover, rice, noodles and their products, breakfast cereals, and roots and tubers are the common staple foods for most Taiwanese people and are treated as everyday habitual foods. To understand the relationships between particular eating behaviors associated with inflammation and glycation, expressed as CRP and HbA1c, respectively, on MCI, these three food groups were excluded from the analysis.

### Study Outcome

In the NAHSIT 2014–2016, the cognitive function was evaluated using the MMSE score in the participants aged ≥65 years. The MMSE includes 30 questions with different perspectives of orientation to time and place, registration, recall, attention, calculation, language, and visuospatial function; the total MMSE score ranges from 0 to 30. Participants with a score ≤ 18 for illiterate individuals, participants with a score ≤ 21 for those with only an elementary education, and participants with a score of ≤ 25 for those with a middle school or higher education were defined as having MCI.

### Statistical Analysis

Complete-case analyses were first conducted to investigate the combined effect of inflammation and glycation with MCI and its associated dietary patterns. Continuous variables are expressed as the mean and SD. Dichotomous variables are presented as numbers and percentages. The Kruskal–Wallis *H* and chi-square tests were performed to compare the differences in the distributions of sample characteristics between groups. Spearman's correlations among MMSE scores, CRP, and HbA1c were calculated. Moreover, the likelihood of MCI was examined using the logistic regression with odds ratios (*OR*s) and 95% *CI*s. In addition, a multivariate logistic regression was used to explore the relationships between a combination of CRP and HbA1c with MCI after adjusting for potential confounders that might cause a predisposition to cognitive decline, which included age, sex, systolic blood pressure (SBP), diastolic blood pressure (DBP), body mass index (BMI), smoking status, alcohol consumption, physical activity, stroke, depression, and sampling strata.

A reduced rank regression (RRR) can be used to derive the dietary patterns for food groups based on the dependent variables of interest in nutritional epidemiology (26). The aim of RRR was to identify the dietary patterns that explain as much response variation as possible by applying the PROC PLS procedure. Therefore, a RRR was performed to calculate two factors (CRP and HbA1C) with their factor loadings based on the predictors of 20 food groups and response variables of CRP and HbA1C. Therefore, the factor loadings for the two factors were separately divided into tertiles: high (T3), medium (T2), and low (T1). The combined effect based on the tertiles for the two factors was then classified into three groups: group 1 with both T3, group 2 with mixed type, and group 3 with both T1. In addition, multivariate logistic regressions were adjusted for the aforementioned associated factors and were conducted to explore the associations of dietary patterns based on the tertiles in combined groups with MCI as well as the specific food group with the two factor loadings >0.2 or < −0.2 and were shown in the same directions.

The imputed datasets to deal with missing values were used to assess the robustness of the study results from complete-case analyses. The fully conditional specification (FCS) multiple imputation techniques by PROC MI and PROC MIANALYZE procedures were performed to avoid the non-response bias with regard to the missing data in CRP, HbA1c, and associated confounders, and five imputations were created. All the statistical analyses were performed using the Statistical Analysis System software (SAS System for Windows, vs. 9.4; SAS Institute, Cary, NC, USA). The statistical significance was defined as *p* < 0.05.

## Results

### Combined Effect of CRP and HbA1c on MCI

In total, 536 participants with complete data were included in the final analysis. They were divided into four groups, IG group (43 subjects), IN group (86 subjects), NG group (93 subjects), and NN group (314 subjects), of which 93 dementia cases were identified.

**Figure 2:**
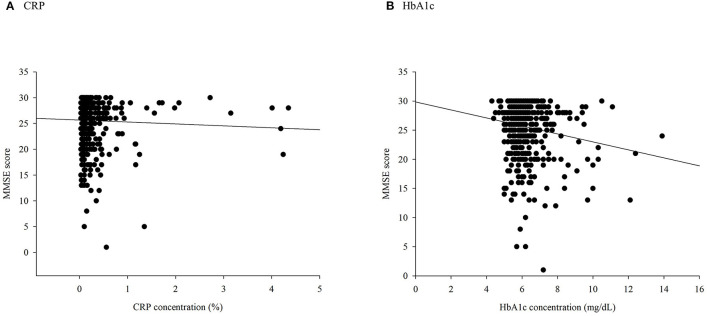
A scatter plot of Mini-Mental State Examination (MMSE) scores based on **(A)** C-reactive protein (CRP) and **(B)** glycated hemoglobin (HbA1c).

There were significant differences in the distribution of demographic characteristics based on the status of combined high levels of CRP or HbA1c, except for SBP (*p* = 0.197), DBP (*p* = 0.192), sex (*p* = 0.460), smoking status (*p* = 0.603), alcohol consumption (*p* = 0.179), stroke (*p* = 0.905), and depression (*p* = 0.170). As shown in Table 1, the IG and IN groups were older (*p* = 0.012) and had lower proportions of variables of physical activity (*p* = 0.002). In addition, a negative correlation between HbA1c and MMSE scores was observed (rho = −0.098, *p* = 0.023), whereas CRP was not negatively correlated with cognitive scores (rho = −0.074, *p* = 0.086, Figure 2).

**Table 1:** The distributions of demographic characteristics according to a combination of C-reactive protein (CRP) and glycated hemoglobin (HbA1c)^a^.

	**Combination of CRP and HbA1c^c^**				***P* value^b^**
	**IG**	**IN**	**NG**	**NN**	
	**(*N* = 43)**	**(*N* = 86)**	**(*N* = 93)**	**(*N* = 314)**	
Age, years, mean ± SD	73.05 ± 5.69	75.13 ± 7.14	71.98 ± 6.03	72.59 ± 5.93	0.012
SBP, mmHg, mean ± SD	135.14 ± 19.58	135.44 ± 17.5	133.26 ± 15.89	132.03 ± 18.55	0.197
DBP, mmHg, mean ± SD	73.77 ± 11.68	76.02 ± 10.39	72.72 ± 9.89	75.22 ± 10.88	0.192
BMI, kg/m 2, mean ± SD	27.21 ± 3.44	25.35 ± 4.05	25.76 ± 3.96	24.35 ± 3.57	<0.001
CRP, mg/dL, mean ± SD	0.65 ± 0.7	0.67 ± 0.84	0.11 ± 0.05	0.1 ± 0.04	<0.001
HbA1c, %, mean ± SD	7.72 ± 1.07	5.85 ± 0.37	7.5 ± 1.37	5.75 ± 0.38	<0.001
Sex, *N* (%)					0.460
Men	21 (48.8)	48 (55.8)	42 (45.2)	167 (53.2)	
Women	22 (51.2)	38 (44.2)	51 (54.8)	147 (46.8)	
Educational level, *N* (%)					0.029
None	6 (14)	10 (11.6)	18 (19.4)	29 (9.2)	
1–8 years	27 (62.8)	47 (54.7)	40 (43)	140 (44.6)	
9–13 years	6 (14)	21 (24.4)	24 (25.8)	90 (28.7)	
14 years and above	4 (9.3)	8 (9.3)	11 (11.8)	55 (17.5)	
Smoking status, *N* (%)					0.603
Yes	4 (9.3)	10 (11.6)	4 (4.3)	31 (9.9)	
Quit	9 (20.9)	17 (19.8)	20 (21.5)	53 (16.9)	
No	30 (69.8)	59 (68.6)	69 (74.2)	230 (73.2)	
Alcohol consumption, *N* (%)					0.179
Yes	10 (23.3)	38 (44.2)	38 (40.9)	141 (44.9)	
Quit	5 (11.6)	6 (7)	6 (6.5)	16 (5.1)	
No	28 (65.1)	42 (48.8)	49 (52.7)	157 (50)	
Physical activity, *N* (%)					0.002
Yes	9 (20.9)	19 (22.1)	37 (39.8)	127 (40.4)	
No	34 (79.1)	67 (77.9)	56 (60.2)	187 (59.6)	
Stroke, *N* (%)					0.905
Yes	2 (4.7)	3 (3.5)	5 (5.4)	12 (3.8)	
No	41 (95.3)	83 (96.5)	88 (94.6)	302 (96.2)	
Depression, *N* (%)					0.170
Yes	3 (7)	1 (1.2)	5 (5.4)	8 (2.5)	
No	40 (93)	85 (98.8)	88 (94.6)	306 (97.5)	

a *SBP, systolic blood pressure; DBP, diastolic blood pressure; BMI, body mass index; SD, standard deviation*.

b *Analyzed using the chi-square test and the Kruskal–Wallis H-Test*.

c *The cutoff points for high level were identified as CRP > 0.21 mg/dl and HbA1c ≥ 6.50%, respectively. IG, participants with high levels of both inflammation and glycation; IN, participants with high levels of inflammation but not glycation; NG, participants without high levels of inflammation but with glycation; NN, participants with high levels of neither inflammation nor glycation*.

As presented in Table 2, the IG group (adjusted *OR* [a*OR*] = 3.51; 95% *CI* = 3.47, 3.55; *p* < 0.001) and the EIG group (a*OR* = 1.61; 95% *CI* = 1.60, 1.62; *p* < 0.001) were associated with higher odds of MCI than the NN group after controlling for age, sex, SBP, DBP, BMI, smoking status, alcohol consumption, physical activity, stroke, depression, and sampling strata in all cases. Moreover, when excluding the effects of the potential confounding factors in the multivariate logistic regression using multiple imputation, the findings of the effect of the IG group and EIG group on the odds of MCI remained largely unchanged (both *p* < 0.001).

**Table 2:** Associations of the combined effect of CRP and HbA1c with mild cognitive impairment (MCI)^a^.

**Combination of CRP and HbA1c^d^**	**Complete cases (*****N*** **=** **536)**			**Multiple imputation (*****N*** **=** **1250)**		
	**OR**	**95% CI**	***P* value^b^, ^c^**	**OR**	**95% CI**	***P* value^b^, ^c^**
IG	3.51	3.47, 3.55	<0.001	2.18	2.16, 2.19	<0.001
EIG	1.61	1.60, 1.62	<0.001	1.23	1.22, 1.23	<0.001
NN	1.00			1.00		
Trend			<0.001			<0.001
IG	3.52	3.48, 3.56	<0.001	2.17	2.15, 2.18	<0.001
IN	1.49	1.47, 1.50	<0.001	1.36	1.35, 1.36	<0.001
NG	1.73	1.71, 1.74	<0.001	1.10	1.09, 1.10	<0.001
NN	1.00			1.00		
Trend			<0.001			<0.001
Interaction (CRP and HbA1c)			<0.001			<0.001

a *OR, odds ratio; CI, confidence interval*.

b *Analyzed using the multivariate logistic regression*.

c *Adjusted for age, sex, systolic blood pressure, diastolic blood pressure, body mass index, smoking status, alcohol consumption, physical activity, stroke, depression, and sampling strata*.

d *The cutoff points for high level were identified as CRP > 0.21 mg/dl and HbA1c ≥ 6.50%, respectively. IG, participants with high levels of both inflammation and glycation; EIG, participants with high levels of either inflammation or glycation; IN, participants with high levels of inflammation but not glycation; NG, participants without high levels of inflammation but with glycation; NN, participants with high levels of neither inflammation nor glycation*.

After the adjustment, the results showed that the highest odds of MCI were found in the IG group (a*OR* = 3.52; 95% *CI* = 3.48, 3.56; *p* < 0.001), followed by the NG group (a*OR* = 1.73; 95% *CI* = 1.71, 1.74; *p* < 0.001), and the IN group (a*OR* = 1.49; 95% *CI* = 1.47, 1.50; *p* < 0.001; Table 2) compared with the NN group from complete-case analysis. In addition, the IG group still showed a significant result with the highest *OR* by multiple imputation (*p* < 0.001).

### Dietary Patterns and Mild Cognitive Dysfunction

In the NAHSIT 2014–2016, 536 participants were included in the reduced rank regression analysis. The dietary patterns explained 2.208 and 1.146% of the variation in the response variables of CRP and HbA1c in the factors 1 and 2, respectively. The factor loadings for food groups with a value of ≥0.2 and ≤ −0.2 are marked in boldface type (Table 3). The findings showed that the food groups for factor 1 were represented by fresh fruits, nuts and seeds, soybean products, processed meat products, and organ meats. In addition, the results showed that the composition of factor 2 greatly differed, with food groups described by vegetables, whole grains, nuts and seeds, flavored milk, soybean products, eggs, fish, poultry, organ meats, and tea. Moreover, the factor loadings of dietary patterns by multiple imputation reported similar findings. In general, in terms of the loadings of factor 1 and factor 2 using complete cases and multiple imputation, the food group of nuts and seeds was expressed in the same directions.

**Table 3:** Factor loadings for the combination of 20 food frequencies for explaining the effects of CRP and HbA1c, respectively, as derived by a reduced rank regression using the data source of the Nutrition and Health Survey in Taiwan (NAHSIT) 2014–2016^a^.

**Food group**	**Factor loading^b^**											
	**Complete cases^c^**		**Imputation 1^d^**		**Imputation 4^d^**		**Imputation 3^d^**		**Imputation 4^d^**		**Imputation 5^d^**	
	**Factor 1**	**Factor 2**	**Factor 1**	**Factor 2**	**Factor 1**	**Factor 2**	**Factor 1**	**Factor 2**	**Factor 1**	**Factor 2**	**Factor 1**	**Factor 2**
Vegetables	−0.0247	**0.2546**	−0.0273	0.0125	−0.1565	0.1307	0.1017	–**0.3230**	−0.1647	**0.2761**	0.0859	–**0.3079**
Fresh fruits	–**0.2736**	0.0113	–**0.3606**	−0.0828	–**0.4494**	−0.0869	−0.1364	–**0.4175**	–**0.5217**	**0.3048**	−0.1488	–**0.4833**
Whole grains	−0.1030	–**0.2936**	−0.0999	–**0.3229**	−0.0321	–**0.3949**	–**0.2204**	**0.2082**	−0.0834	–**0.2379**	−0.1770	0.1575
Nuts and seeds	–**0.5771**	–**0.2496**	–**0.4767**	−0.0378	–**0.5988**	−0.1783	–**0.2848**	−0.1995	–**0.5537**	0.0222	–**0.5419**	–**0.3155**
Dairy products	−0.1255	−0.1165	−0.1166	−0.1056	−0.0938	−0.1927	−0.1721	0.0121	−0.1091	−0.0892	−0.1413	0.0052
Flavored milk	−0.1251	–**0.2133**	−0.0700	−0.0711	0.1899	–**0.2815**	–**0.2333**	0.0058	−0.0752	−0.1014	–**0.2336**	0.0399
Soybean products	**0.2362**	**0.4090**	0.1776	**0.3788**	−0.0175	**0.4813**	0.1429	–**0.2434**	0.0291	**0.3464**	**0.2225**	−0.1818
Eggs	−0.0169	–**0.2186**	0.1359	–**0.3357**	0.0139	0.0814	−0.0415	**0.3281**	0.1171	**0.3622**	0.0446	−0.0972
Fish	−0.0879	**0.2118**	−0.0804	0.0377	−0.1153	–**0.2252**	–**0.2564**	0.0252	−0.0609	0.0177	–**0.2624**	0.0362
Shellfish	−0.0555	0.0378	−0.1204	−0.0429	−0.1117	−0.1428	−0.1531	−0.0487	−0.1146	−0.0431	−0.1259	−0.0426
Seafood products	−0.0229	0.1028	0.0130	0.1054	**0.2317**	0.0097	0.1498	−0.0159	0.1756	0.1845	**0.6119**	−0.1678
Poultry	0.1883	–**0.3813**	**0.3551**	–**0.4056**	**0.2678**	–**0.4629**	0.0134	**0.3679**	**0.2921**	–**0.3257**	0.0400	**0.5315**
Red meat	0.0481	−0.1367	**0.2123**	–**0.2866**	0.0185	0.0044	0.0626	0.1497	−0.0234	−0.0422	0.1121	0.1618
Processed meat products	**0.2607**	−0.1494	**0.5103**	0.0531	**0.2227**	−0.0064	**0.6897**	**0.2260**	0.1511	−0.0479	−0.0756	**0.2102**
Organ meats	**0.5130**	–**0.3413**	−0.1379	−0.1491	−0.0459	0.0216	−0.1461	0.0179	−0.0214	−0.0468	0.0107	−0.0905
Fried foods	0.1988	−0.1022	**0.2537**	−0.1979	**0.2940**	−0.0673	**0.2318**	–**0.3616**	0.1979	**0.4755**	0.0260	0.1935
Snacks	−0.1955	−0.1799	0.0580	−0.1497	0.0058	0.1319	−0.0784	−0.1801	–**0.3132**	0.0820	0.0144	−0.1427
Coffee	−0.1613	0.0734	−0.0604	**0.3609**	–**0.2066**	0.0907	0.0247	–**0.2693**	−0.1053	**0.2907**	0.0301	−0.1367
Tea	−0.0965	–**0.2978**	−0.1174	–**0.2451**	−0.0654	–**0.3358**	–**0.2190**	0.1310	−0.1076	−0.1853	−0.1777	0.0970
Sweetened beverages	−0.0085	0.1256	0.0817	–**0.2773**	−0.1787	0.0637	−0.1183	−0.0766	−0.1957	−0.0641	−0.0834	−0.1580
Explained variation, %												
Predictor	5.0904	5.3282	5.8786	5.3325	5.7821	5.5308	4.8724	5.6083	5.9934	5.8051	5.1242	6.0410
Dependence	2.2079	1.1459	1.0574	0.3819	0.9530	0.3092	0.8415	0.3629	1.0903	0.6453	1.1421	0.6157

*NAHSIT, Nutrition and Health Survey in Taiwan*.

b *The food groups were used as predictors and the markers of CRP and HbA1c were observed to be dependent; the factor loadings are marked in bold with values >0.2 or < -0.2*.

c *This dietary pattern explained 10.42% of the total food frequency questionnaire (FFQ) variance and explained 3.35% of the CRP and HbA1C variance*.

d *This dietary pattern explained 11.21, 11.31, 10.48, 11.80, and 11.17% of the total FFQ variance, respectively, and 1.44, 1.26, 1.20, 1.74, and 1.76% of the CRP and HbA1C variance for imputation 1, imputation 2, imputation 3, imputation 4, imputation 5, respectively*.

Table 4 presents the relationships of tertiles for both factor loadings and the combined effects of factor loadings of dietary patterns on MCI, both in complete cases and multiple imputation. After adjusting for age, sex, sampling strata, and associated confounders, the *OR*s for MCI were higher in T3 (a*OR* = 1.38; 95% *CI* = 1.37, 1.39; *p* < 0.001) and T2 (a*OR* = 2.13; 95% *CI* = 2.11, 2.15; *p* < 0.001) of the factor loadings in factor 1 compared with T1. Similar results were found for factor 2, which exhibited the highest *OR* for MCI in T2 with statistically significant trend tests (all *p* < 0.001). Concerning the combined effect of tertiles among factor 1 and factor 2, group 1 with both T3 was associated with increased *OR* for MCI (a*OR* = 1.49; 95% *CI* = 1.47, 1.51; *p* < 0.001), while the results of group 2 with a mixed type also presented a higher a*OR* of 1.64 (95% *CI* = 1.62, 1.65; *p* < 0.001), compared with group 3 with both at T1 after considering factors that might affect the relationship. Moreover, slightly different results from multiple imputation were obtained. The group 1 with both in T3 had the highest *OR* for MCI (a*OR* = 4.38; 95% *CI* = 4.34, 4.42; *p* < 0.001; Table 4).

**Table 4:** Odds ratios and 95% *CI*s for mild cognitive impairment, by tertiles of the dietary pattern and the combined effect^a^.

**Dietary pattern explained by CRP and HbA1c**	**Complete cases (*****N*** **=** **536)**			**Multiple imputation (*****N*** **=** **1,250)**		
	**OR**	**95% CI**	***P* value^b^, ^c^**	**OR**	**95% CI**	***P* value^b^, ^c^**
Factor 1						
T3	1.38	1.37, 1.39	<0.001	2.03	2.02, 2.04	<0.001
T2	2.13	2.11, 2.15	<0.001	2.38	2.37, 2.40	<0.001
T1	1.00			1.00		
Trend			<0.001			<0.001
Factor 2						
T3	1.90	1.88, 1.92	<0.001	1.93	1.93, 1.94	<0.001
T2	2.73	2.71, 2.76	<0.001	1.25	1.24, 1.25	<0.001
T1	1.00			1.00		
Trend			<0.001			<0.001
Combination of factor 1 and factor 2						
Group 1 (both in T3)	1.49	1.47, 1.51	<0.001	4.38	4.34, 4.42	<0.001
Group 2 (mixed type)	1.64	1.62, 1.65	<0.001	2.67	2.65, 2.69	<0.001
Group 3 (both in T1)	1.00			1.00		
Trend			<0.001			<0.001

a *OR, odds ratio; CI, confidence interval; T1, first tertile; T2, second tertile; T3, third tertile*.

b *Analyzed using the multivariate logistic regression*.

c *Adjusted for age, sex, systolic blood pressure, diastolic blood pressure, body mass index, smoking status, alcohol consumption, physical activity, stroke, depression, and sampling strata*.

**Figure 3:**
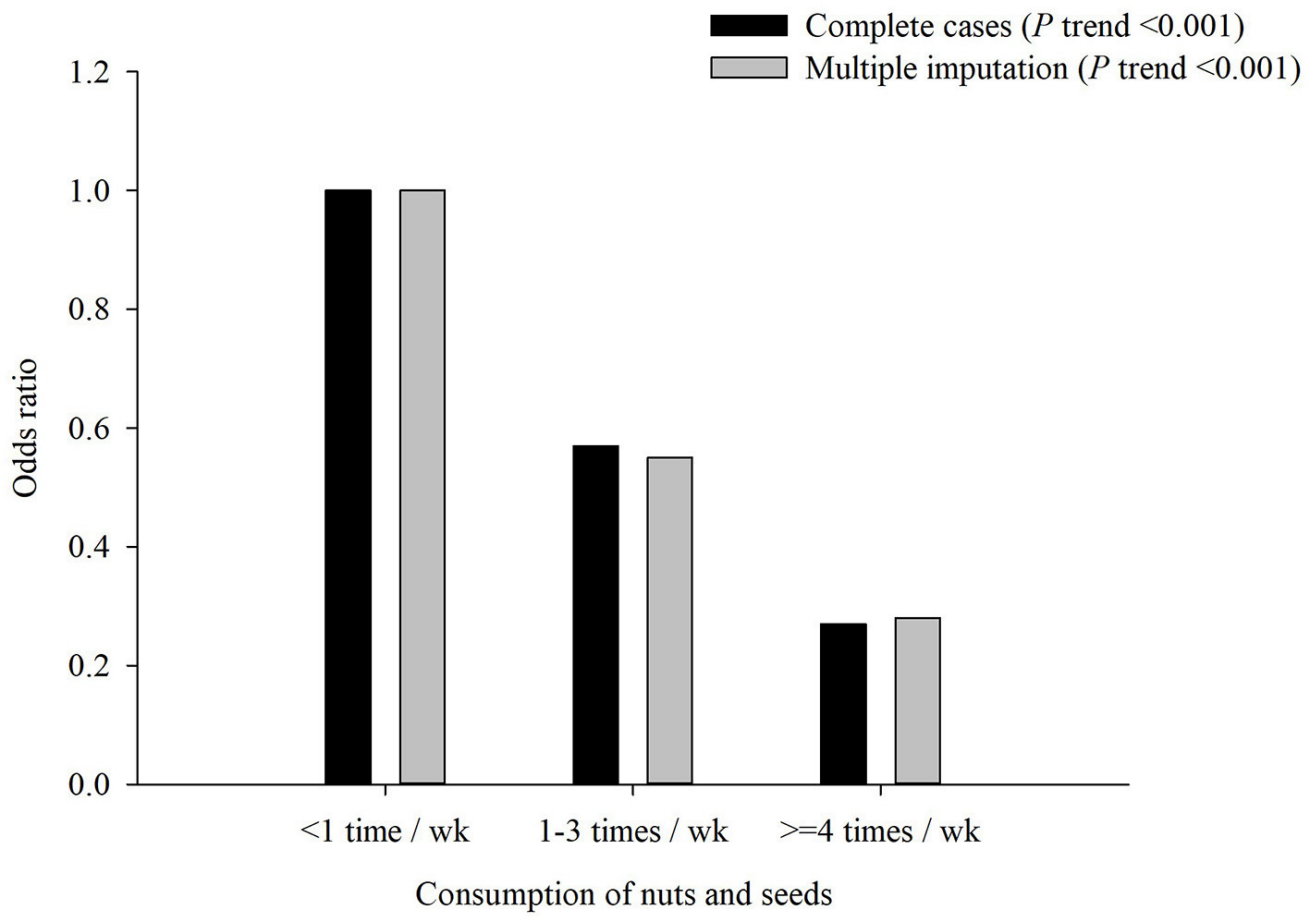
Adjusted odds ratio (aOR) of mild cognitive impairment (MCI) by consumption of nuts and seeds. Test for trend: *p* < 0.001.

Regarding single food groups, the results from the factor loadings presented the same pattern for both factors, but negative factor loadings for CRP and HbA1c were revealed for older adults who consumed higher frequencies than those of other food groups, such as nuts and seeds. Furthermore, significantly decreasing trends (all *p* < 0.001) and protective effect in the occurrence of MCI were observed with an increased intake frequency of nuts and seeds (four or more times per week: a*OR* = 0.27, *p* < 0.001; one to three times per week: a*OR* = 0.57, *p* < 0.001; Figure 3).

## Discussion

Using a nationwide population-based survey, our results demonstrated that the integration of high levels of inflammation, such as CRP, and hyperglycemia, such as HbA1c, showed higher odds of MCI. The dietary patterns related to inflammation and hyperglycemia were associated with MCI. Therefore, better cognitive performance should be a benefit due to an increased consumption of nuts and seeds.

To the best of our knowledge, this is the first study to explore the associations between a combination of inflammation and hyperglycemia and the related dietary patterns with cognitive dysfunction. Previous research only focused on the relationship between the combination of CRP and HbA1c with subsequent outcomes, such as carotid atherosclerosis progression (15), cardiovascular risk (16), severe coronary artery disease (17), and DM (27). Previous epidemiologic studies reported that hyperglycemia, diabetes status, and higher CRP levels were independently associated with the incidence of dementia (28, 29).

Inflammation, as assessed by hs-CRP, was demonstrated to reduce the cognitive function through an acute phase affecting tissue damage (30) by stimulating the deposition of amyloid β (31) or developing cerebral atherosclerosis that can harm the brain function (32). Hyperglycemia is associated with worse cognitive performance, resulting from insulin dysregulation (33), expression of insulin-degrading enzymes (34), and severe hypoglycemic events (35) that might damage the structure of the brain. Furthermore, increased CRP production in the liver might be related to abnormal glucose metabolism (36). Advanced cognitive decline possibly being affected by the combination of these two biomarkers might be a possible explanation.

A recent study of the NAHSIT 2014–2016 examined the dietary patterns related to cognitive function using a RRR (21). It should be noted that the classification of the dietary patterns was based on the MMSE score, not on the biomarkers of inflammation and glycation. Better MMSE scores were correlated with the dietary patterns of higher intake of some foods, such as fresh fruits, nuts and seeds, whole grains, seafood products, and fish. However, the influence of diet on glycemic disorders or inflammation has not been discussed, which might play an important role in the cognitive dysfunction. Moreover, the participants without CRP and HbA1c were excluded, resulting in a smaller sample size than the aforementioned study of the same survey years.

In our study, we alternatively used CRP and HbA1c as the response variables. Subsequently, the tertiles of factor scores related to HbA1c and CRP were treated as the predictors of MCI. Some foods have been reported to be related to hyperglycemia (37–39). Several studies have focused on an inflammatory diet (40), a cognitive diet (21), and a Mediterranean-type diet (41). However, no study has simultaneously discussed the diets corresponding to both CRP and HbA1c; additionally, our observations highlight the importance of nuts and seeds by considering a reduction in glycemic metabolism and inflammation. Furthermore, although the tertiles of T2 in factor 1 and factor 2 showed the highest risk in complete-case analyses, group 1 with both in T3 reached statistical significance with highest *OR*s when the findings were obtained by multiple imputation. A possible explanation for this result might be the small sample size.

Our study indicated that the consumption of more nuts and seeds decreased the odds of cognitive dysfunction. The higher intake of nuts and seeds were associated with reduced inflammatory levels, which might be explained by the inverse relationship of nut consumption with risks of metabolic syndrome, cardiovascular disease, and diabetes (42). Moreover, the dietary consumption of nuts has beneficial effects on inflammation and glucose control in patients with DM (43). An explanation of the mechanism for maintaining better cognitive performance might be folate (44), which can reduce the risk of dementia (45, 46).

Our study has several strengths. First, to the best of our knowledge, no previous studies have discussed the association between inflammation and hyperglycemia associated with MCI. Second, the correlation of the dietary patterns associated with CRP and HbA1c levels was assessed in this study. Therefore, the combination of CRP and HbA1c levels and dietary patterns on cognitive function was simultaneously addressed. Third, the data source of NAHSIT 2014–2016 was a community-based survey. Thus, this study provides high representativeness. Moreover, the robustness of our study was confirmed by using a much larger sample size when generalizing the findings compared with other analyses in Western countries (47, 48).

However, there are several limitations that should be discussed. First, the participants were obtained from community-based samples. Thus, the questionnaires in NAHSIT 2014–2016 might be subject to recall bias. Second, the patients with MCI were identified using MMSE scores in this population-based survey. The identification of MCI might have been underestimated because of the characteristics of the education level, in which literacy was used to define the cognitive decline. Third, causal inferences could not be examined in the relationship between dietary patterns and MCI as this was a cross-sectional study. Finally, there were several important biomarkers of inflammation and hyperglycemia that were not available in NAHSIT 2014–2016, such as inflammatory cytokines, leukocyte ratio, monocyte ratio, and 2-h post-load plasma glucose during the oral glucose tolerance test. Moreover, although fasting plasma glucose could be used in this community-based survey, the purpose of our study was to evaluate the association between long-term glycemic control over 2–3 months and current dietary patterns by using HbA1c as a biomarker of hyperglycemia.

In conclusion, the results of this nationwide population-based survey showed that the combined effect of high levels of inflammation and hyperglycemia was associated with increased odds of MCI, which was true for a high level of only CRP or a high level of only HbA1c. Furthermore, a positive relationship between the dietary patterns and inflammation and hyperglycemia with MCI was found in this cross-sectional study, while eating nuts and seeds was associated with better cognition.

## Data Availability Statement

The data described in the article, code book, and analytic code will not be made available because the data source used in this study was managed by Health Promotion Administration. The researchers were required to submit an application to use the data for scientific purposes, and these data were not publicly accessed.

## Ethics Statement

The studies involving human participants were reviewed and approved by The Joint Institutional Review Board of Taipei Medical University and Institutional Review Board on Biomedical Science Research, Academia Sinica, Taiwan. The patients/participants provided their written informed consent to participate in this study.

## Author Contributions

Y-CF conceived the idea, performed the statistical analysis, and drafted the manuscript. C-CC, BB, and W-HP contributed to providing clinical knowledge and reviewing the manuscript. C-HB reviewed and revised the idea and study design, supported the grants, and helped to edit the manuscript. Y-CF and C-HB are the guarantors of this work to take responsibility for this study. All authors read and approved the final manuscript.

## Funding

This work was funded by the Health Promotion Administration, Ministry of Health and Welfare (MOHW109-HPA-H-114-144702; D1060103) and the Ministry of Science and Technology of Taiwan (MOST-103-2314-B038-033-MY3).

## Conflict of Interest

The authors declare that the research was conducted in the absence of any commercial or financial relationships that could be construed as a potential conflict of interest.

## Publisher's Note

All claims expressed in this article are solely those of the authors and do not necessarily represent those of their affiliated organizations, or those of the publisher, the editors and the reviewers. Any product that may be evaluated in this article, or claim that may be made by its manufacturer, is not guaranteed or endorsed by the publisher.
